# Cyclic nucleotide signaling as a drug target in retinitis pigmentosa

**DOI:** 10.1002/1873-3468.70107

**Published:** 2025-07-09

**Authors:** Katri Vainionpää, Ahmed B. Montaser, Henri Leinonen

**Affiliations:** ^1^ School of Pharmacy, Faculty of Health Sciences University of Eastern Finland Kuopio Finland

**Keywords:** cAMP, cGMP, cyclic nucleotides, retinal degeneration, retinitis pigmentosa

## Abstract

Retinitis pigmentosa (RP) is a heterogeneous group of inherited retinal degenerative diseases caused by mutations in over 90 genes. The complexity of its genetic background and economic barriers limit the broad application of targeted gene therapies. Therefore, general pharmacological strategies to slow disease progression, regardless of the underlying mutation, are needed. Cyclic nucleotide second messengers, such as cyclic guanosine monophosphate (cGMP) and cyclic adenosine monophosphate (cAMP), are important for normal retinal function. This includes phototransduction, for which cGMP signaling is essential. Dysregulation of the cyclic nucleotide systems is associated with retinal degeneration, and the inhibition of cGMP or cAMP signaling has shown beneficial effects in several retinal degeneration disease models. Here, we propose these systems as drug targets for RP.

**Impact statement**

This perspective proposes targeting cyclic nucleotide signaling (cGMP and cAMP) as a mutation‐independent therapeutic strategy for retinitis pigmentosa, offering broad potential for disease‐modifying treatment potentially through drug repurposing and novel drug delivery systems.

## Graphical abstract

Disruptions in cGMP and cAMP signaling can contribute to retinal dysfunction and photoreceptor loss in retinitis pigmentosa. This perspective examines the mechanisms and evaluates emerging evidence on targeting these pathways as a potential therapeutic strategy to slow or prevent retinal degeneration.

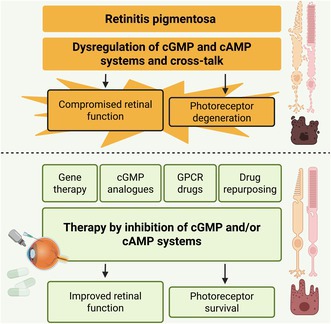

## Abbreviations

**5‐HTR**, serotonergic receptor

**ADCY**, adenylate cyclase

**AIF**, apoptosis‐inducing factor

**AIPL1**, aryl‐hydrocarbon‐interacting protein‐like 1

**AMP**, adenosine monophosphate

**ATF4**, activating transcription factor 4

**ATP**, adenosine triphosphate

**BAD**, Bcl‐2‐associated agonist of cell death

**BRB**, blood–retinal barrier

**CaM**, calmodulin

**CAMK**, calcium/calmodulin‐dependent protein kinase

**cAMP**, cyclic adenosine monophosphate

**CCAAT**, CCAAT‐enhancer‐binding proteins

**cGMP**, cyclic guanosine monophosphate

**CHOP**, CCAAT/enhancer‐binding protein homologous protein

**CNG**, cyclic nucleotide‐gated

**CREB**, cAMP response element‐binding protein

**DAG**, diacylglycerol

**DNA**, deoxyribonucleic acid

**DR**, dopamine receptor

**eIF2**, eukaryotic initiation factor 2

**eNOS**, endothelial nitric oxide synthase

**EPAC**, exchange protein activated by cAMP

**ER**, endoplasmic reticulum

**ERG**, electroretinogram

**ERK**, extracellular signal‐regulated kinase

**GC**, guanylyl cyclase

**GCAPs**, guanylate cyclase activating proteins

**GMP**, guanosine monophosphate

**GPCR**, G protein‐coupled receptor

**GTP**, guanosine triphosphate

**GUCY**, guanylate cyclase

**HDAC**, histone deacetylase

**IFT88**, intraflagellar transport protein 88 homolog

**IMPDH**, inosine monophosphate dehydrogenase

**IP**, intraperitoneal

**IP3**, inositol 1,4,5‐trisphosphate

**IVI**, intravitreal injection

**MAPK**, mitogen‐activated protein kinases

**MEK**, mitogen‐activated protein kinase

**MMF**, mycophenolate mofetil

**NCKX**, potassium‐dependent sodium‐calcium exchangers

**NF‐κB**, nuclear factor kappa‐light‐chain‐enhancer of activated B cells

**ovl**, zebrafish mutant oval

**P23H**, proline conversion to histidine at position 23 of Rhodopsin gene

**PARP**, poly(ADP‐ribose) polymerase

**PDE**, phosphodiesterase

**PFKFB3**, 6‐phosphofructo‐2‐kinase/fructose‐2,6‐bisphosphatase 3

**PI3K**, phosphoinositide 3‐kinases

**PIP2**, phosphatidylinositol 4,5‐bisphosphate

**PKA**, protein kinase A

**PKC**, protein kinase C

**PKG**, protein kinase G

**PLC**, phospholipase C

**PRKG1**, cGMP‐Dependent Protein Kinase 1

**PRPH2**, peripherin 2

**RAF1**, Ser/Thr kinase

**RAS**, rat sarcoma virus oncogene

**RD**, retinal degeneration

**rd1**, retinal degeneration 1 mouse model

**rd10**, retinal degeneration 10 mouse model

**rd2**, retinal degeneration 2 mouse model

**RHO**, rhodopsin

**RLBP1**, retinaldehyde binding protein 1

**RNA**, ribonucleic acid

**ROCK**, Rho‐associated coiled‐coil kinase

**RP**, Retinitis pigmentosa

**RPE**, retinal pigment epithelium

**RPE65**, retinoid isomerohydrolase RPE65

**RPGR**, retinitis pigmentosa GTPase regulator

**RyR2**, ryanodine receptor 2

**SC**, subcutaneous

**TOP2A**, DNA topoisomerase 2‐alpha

**USH2A**, usherin

**VEGF**, vascular endothelial growth factor

**αR**, adrenergic alpha receptor

**βR**, adrenergic beta receptor

Retinitis pigmentosa (RP) is a group of currently untreatable inherited retinal dystrophies characterized by progressive degeneration of rod photoreceptors, initially leading to loss of night and peripheral vision [1, 2]. With disease progression, cone photoreceptors start to degenerate as well, resulting in a gradual loss of visual function required for daily activities and potentially complete blindness over time. The onset of symptoms can vary widely, with some individuals experiencing initial signs in childhood, whereas most patients notice symptoms later in life, typically in their 30s or 40s. The global prevalence of RP is estimated to be approximately one in 4000 individuals, although this figure can vary based on geographic and ethnic factors [1]. Studies generally suggest a prevalence ranging from one in 2000 to one in 7000, indicating a significant degree of variability across different populations [2, 3]. The inheritance patterns of RP are diverse, with approximately 30–40% of cases being autosomal dominant, 50–60% autosomal recessive, and 5–15% X‐linked [1]. To date, mutations in at least 90 distinct genes are linked to RP [4]. RP significantly impacts the quality of life of working‐age individuals and causes significant social and economic burden in society [5].

As a genetic disease that can be treated *via* locally administered therapeutics (directly into the eye), RP is highly amenable to gene therapy. The first gene therapy, voretigene neparvovec‐rzyl (Luxturna^®^, Spark Therapeutics, Inc., Philadelphia, USA), for inherited retinal degenerations received marketing approval from the US Food and Drug Administration in 2017 for the treatment of biallelic retinoid isomerohydrolase RPE65 (*RPE65*) mutation causing Leber congenital amaurosis and RP [6]. Currently, clinical trials for targeted gene therapies are registered or ongoing for cyclic nucleotide‐gated channel subunit alpha 1 (*CNGA1*, [7]) phosphodiesterases 6a and ‐b (*PDE6A*, *PDE6B*, [8, 9]), retinaldehyde binding protein 1 (*RLBP1*, [10]), rhodopsin (RHO, [8, 11, 12]), X‐linked retinitis pigmentosa GTPase regulator (*RPGR*, [13, 14, 15, 16, 17, 18, 19, 20, 21, 22]), and usherin (*USH2A*, [23]) mutations. However, several factors, such as the heterogeneity of the genetic background of RP, the relatively low number of patients with the same RP mutation, lack of molecular genetic diagnostics for idiopathic RP cases, and the high price of targeted gene therapies, limit the large‐scale application of gene therapies in the whole spectrum of RP forms in the foreseeable future. This rationalizes the research and development of mutation‐agnostic treatment options along with targeted gene therapies. Furthermore, general treatments that are effective in RP could potentially mitigate other retinal degenerative diseases that share similar cell stress mechanisms, such as inflammation, oxidative and metabolic stress, and calcium imbalance (reviewed in [24, 25]). Two attractive and readily targetable molecular pathways involved in retinal degeneration are the cyclic guanosine monophosphate (cGMP) and cyclic adenosine monophosphate (cAMP) signaling pathways [26, 27, 28, 29, 30, 31, 32, 33]. Here, we discuss cGMP and cAMP signaling as plausible therapeutic targets for decelerating retinal degeneration in RP.

## The cGMP pathway as a drug target in retinitis pigmentosa

**Fig. 1:**
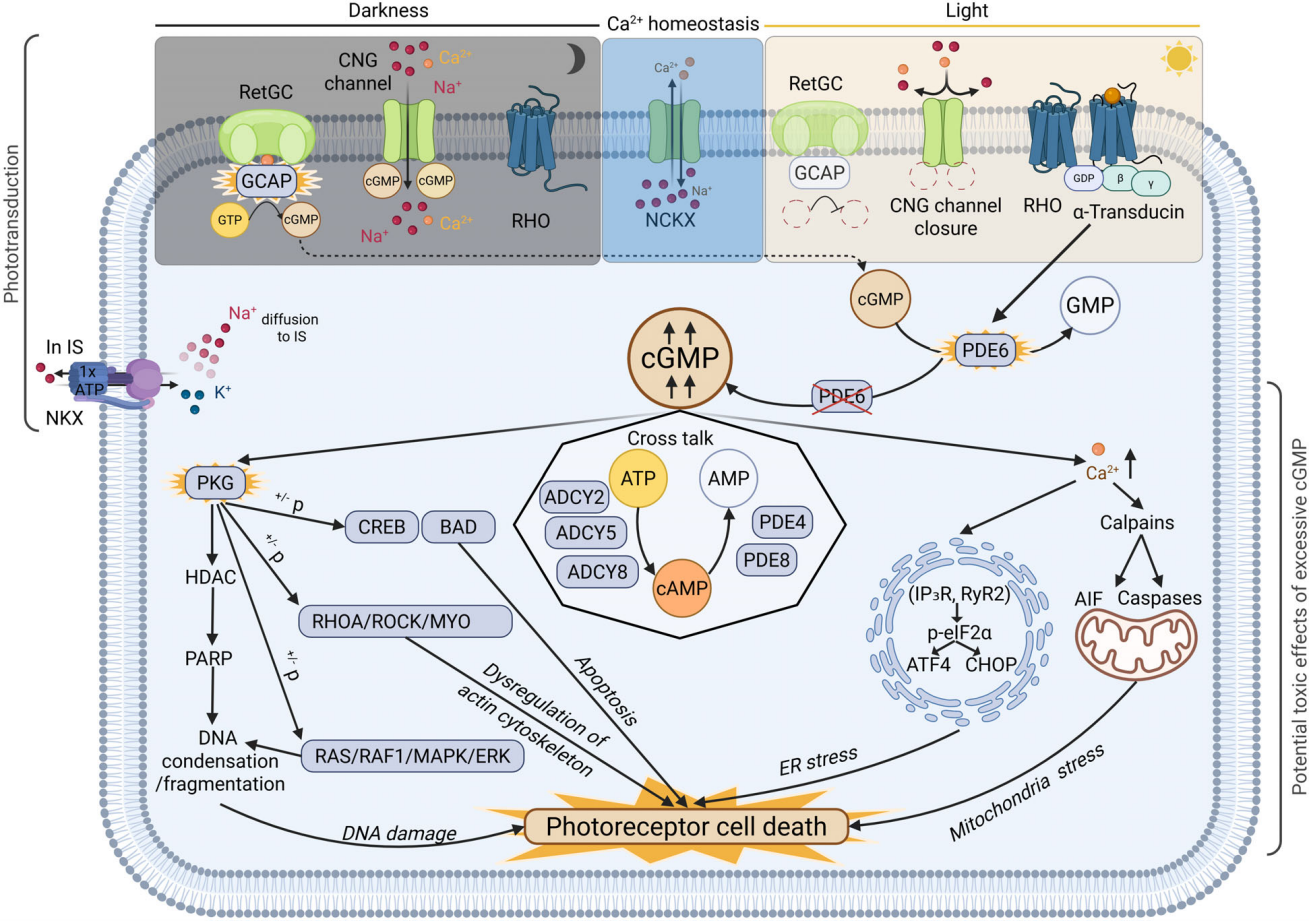
cGMP is required for phototransduction, but excessive levels promote cell death in photoreceptors. Normal physiology: In darkness, intracellular cGMP binds to cyclic nucleotide‐gated channels (CNG) to allow the continuous influx of Na^+^ and Ca^2+^ ions into the photoreceptor, a phenomenon known as the ‘dark current’ [34]. Upon light exposure, the light‐sensitive opsin in the photoreceptor (RHO in rods) becomes activated, leading to the activation of the heterotrimeric G protein transducin. Transducin then activates phosphodiesterase 6 (PDE6), which degrades excess cGMP and causes the closure of CNG channels, resulting in photoreceptor hyperpolarization. Phototransduction is controlled by a sodium‐calcium exchanger (NCKX1 in rods, NCKX2 in cones) located in the photoreceptor outer segment, and a Na^+^/K^+^‐ATPase pump in the inner segment. cGMP levels are also regulated by membrane‐bound guanylyl cyclases (GCs) that are activated by the calcium‐sensitive guanylate cyclase activating proteins (GCAPs) leading to cGMP synthesis. In disease: Persistent cGMP elevation can occur in some disease conditions, such as PDE6 mutations, leading to overactivation of cell signaling pathways that promote photoreceptor death. For instance, increased intracellular calcium can hyperactive the Ca^2+^‐activated cysteine proteases (calpains) that leads to mitochondrial stress *via* caspase‐dependent or independent mechanisms [through e.g., the release of pro‐apoptotic proteins such as apoptosis‐inducing factor (AIF)] [25]. Increased intracellular calcium also promotes further Ca^2+^ release from the endoplasmic reticulum (ER) by activating inositol 1,4,5‐trisphosphate receptors (IP3Rs) and ryanodine receptor 2 (RyR2). This triggers ER stress through phosphorylation of eukaryotic initiation factor 2 alpha (eIF2α), leading to the activation of pro‐apoptotic proteins such as activating transcription factor 4 (ATF4) and CCAAT/enhancer‐binding protein homologous protein (CHOP), ultimately resulting in chronic ER stress and cell death. Another key feature of persistent cGMP signaling is the chronic activation of protein kinase G (PKG), which is thought to disrupt the phosphorylation of neuroprotective proteins such as cAMP response element‐binding protein (CREB) and Bcl‐2‐associated agonist of cell death (BAD), ultimately leading to photoreceptor apoptosis [35, 36]. PKG overactivation has also been associated with mislocalization of the small GTPase RhoA, activation of Rho‐associated coiled‐coil kinase (ROCK), and dysregulation of myosin, collectively contributing to actin cytoskeleton instability and cell death. Furthermore, PKG may interfere with the extracellular signal‐regulated kinase (ERK) pathway by altering the activity of intermediate proteins such as rat sarcoma virus oncogene (RAS), Ser/Thr kinase (RAF1), and mitogen‐activated protein kinases (MAPK), thereby promoting apoptosis. The activation of histone deacetylase (HDAC) and poly(ADP‐ribose) polymerase (PARP) observed in retinal degeneration has also been linked to PKG overactivity, suggesting a role in DNA damage and the eventual loss of photoreceptor cells. Created in BioRender. Leinonen, H. (2025) https://BioRender.com/b23yl61.

In rod and cone photoreceptor cells of the retina, cGMP is a critical second messenger in phototransduction, regulating the cyclic nucleotide‐gated (CNG) ion channels to control photoreceptor membrane currents [34]. Under physiological conditions, cGMP levels in the photoreceptor outer segments are tightly regulated by the balanced activities of guanylate cyclases and PDE6. In the dark, elevated cGMP levels keep CNG channels open, enabling a steady influx of sodium and calcium ions (Fig. 1, top part). Upon exposure to light, photopigments undergo a conformational change that activates PDE6. Active PDE6 leads to rapid hydrolysis of cGMP, closure of CNG channels, and hyperpolarization of the photoreceptor cell, ultimately transmitting the visual signal to downstream retinal neurons. This tightly regulated cGMP turnover is essential for a normal visual function. In addition to phototransduction, cGMP also influences various other physiological processes in the retina. For instance, cGMP is implicated in the regulation of fluid transport across the retinal pigment epithelium (RPE), which is essential for preventing retinal edema and maintaining retinal attachment to underlying tissues [37, 38]. However, excessive cGMP under certain pathological conditions can lead to the activation of cell death pathways (see [39], for a comprehensive review).

Elevated cGMP levels are strongly linked to retinal degeneration in *PDE6*‐mutation‐associated RP, where impaired cGMP hydrolysis leads to pathological cGMP accumulation [40]. In addition to the direct detrimental effects of intracellular calcium overload caused by excessive cGMP, downstream harmful events include aberrant phosphorylation of multiple cellular substrates, disruption of cellular homeostasis, and activation of stress‐related signaling pathways that promote photoreceptor cell death (Fig. 1, bottom part; see also [39, 41], for comprehensive reviews). However, targeting cGMP signaling as a therapeutic strategy in RP extends beyond mutations in the *PDE6* gene. Several lines of evidence indicate that various forms of RP, characterized by different genetic mutations, also lead to dysregulated cGMP signaling, suggesting a broader applicability of therapies targeting cGMP [30, 31, 32, 33]. Elevated cGMP levels are implicated in inherited retinal degenerations arising from mutations in genes not only involved directly in its metabolism [e.g., *PDE6B*, aryl‐hydrocarbon‐interacting protein‐like 1 (*AIPL1*), *CNGA1*, CNG channel subunit beta *1* (*CNGB1*), guanylate cyclase 2D (*GUCY2D*), and guanylate cyclase 1 soluble subunit alpha 1 (*GUCY1A*)] but also those involved in supporting photoreceptor integrity and phototransduction processes such as *RHO*, *RPE65*, and peripherin *2* (*PRPH2*). In line with this, recent proteomics data suggest that cyclic nucleotide pathways (both cGMP and cAMP) are severely dysregulated in *Pde6b* ^rd10^ (rd10) mouse retinas with PDE6B deficiency, as well as in *Rho* ^P23H^ (P23H) mutant mouse retinas [33].

Restricting cGMP accumulation and its downstream effects has been beneficial in reducing retinal degeneration in preclinical disease models. Using RNA interference, Tosi *et al*. [42] demonstrated that silencing *Gucy2e*—which resynthesizes cGMP after light activation—or *Cnga1*, which encodes the α‐subunit of the CNG channel, in *Pde6b* ^H620Q^ mice with partial PDE6 loss‐of‐function, mitigated photoreceptor degeneration, as evidenced by preserved outer nuclear layer thickness and improved scotopic a‐wave responses. To corroborate the causality between elevated cGMP and photoreceptor death, Sahaboglu *et al*. [43] demonstrated that selective PDE6 inhibition leading to cGMP elevation induced photoreceptor cell death in wild‐type retinal explants, mimicking the degeneration observed in *Pde6b* ^rd1^ (rd1) mouse retinas. Another promising strategy involves reducing cGMP synthesis by targeting inosine monophosphate dehydrogenase (IMPDH) using mycophenolate mofetil (MMF) [44], thereby inhibiting *de novo* guanine nucleotide production. MMF has been shown to effectively lower intracellular cGMP levels in photoreceptors, providing robust therapeutic effects in early stages of the disease in rd1 and rd10 mouse models of RP by protecting retinal structure and function. In addition, it has been demonstrated that decreased cGMP levels by dopamine ablation protect photoreceptors in cultured rd1 mouse retinas, although the effect on cGMP accumulation was only partial [45]. The therapeutic potential of cGMP inhibition is further supported by evidence obtained from gene therapy studies. For instance, gene therapy approaches have shown promise in restoring normal cGMP signaling in animal models of CNGB1‐ [32] and PDE6B‐associated RP [46], leading to improved photoreceptor function and survival.

Calcium channel blockers, such as diltiazem, nilvadipine, and nicardipine—clinically used as cardiovascular drugs—can mitigate photoreceptor loss in rd1 mice [47, 48]. Nilvadipine was also shown to delay the progression of visual sensitivity loss in the central 10° of the visual field, but not in the central 2° field, in RP patients [49, 50]. The link between calcium influx and photoreceptor death in *Pde6* mutants has been confirmed with genetic studies as *Cngb1* ^−/−^ knockout incorporated into *Pde6g* ^−/−^ and rd10 background delayed retinal degeneration in these mice [51, 52]. This supports the notion that cGMP accumulation, due to impaired PDE6 activity, leads to sustained CNG channel opening and excessive calcium influx, jointly contributing to photoreceptor cell death. However, negative findings have also been reported (reviewed in [53]), as, for instance, diltiazem treatment failed to attenuate retinal degeneration in dogs with the same PDE6B defect as found in rd1 mice [54].

It is notable that chronically elevated cGMP levels continuously activate protein kinase G (PKG), which also promotes photoreceptor cell death [51]. PKG is a downstream effector of cGMP that phosphorylates several proteins relevant to retinal degeneration (Fig. 1). The activation of the PKG pathway was found to trigger photoreceptor death in the retina by disrupting ion balance (*via* potassium/glutamate receptors), impairing survival signaling *via* cAMP response element‐binding protein 1 (CREB1), compromising DNA repair *via* DNA topoisomerase 2‐alpha (TOP2A), and altering metabolic homeostasis *via* 6‐phosphofructo‐2‐kinase/fructose‐2,6‐bisphosphatase 3 (PFKFB3) [35, 55]. Two distinct genes encoding PKGs exist: PKG I (*PRKG1*) and PKG II (*PRKG2*). In addition, two alternatively spliced exons encode the N‐terminal region of PKG I, giving rise to the PKG Iα and PKG Iβ isoforms [56]. According to Ekström *et al*. [57], PKG I is predominantly expressed in photoreceptor outer segments, whereas PKG II shows fainter immunohistochemical expression in inner segments, outer plexiform layer, inner nuclear layer, and the ganglion cell layer of the mouse retina. Distinct functional roles for the two PKG I isoforms in photoreceptors remain unclear; however, some evidence suggests that PKG Iβ is the more abundant splice variant in the mouse eyes [58], but on the other hand, PKG Iα may have a higher affinity for cGMP [59].

Dampening the cGMP pathway by inhibitory cGMP analogues offers a unique opportunity for photoreceptor protection in RP. A medicinal chemistry approach by replacing a nonbridging oxygen in the phosphate backbone of cGMP with sulfur forms phosphorothioate bonds. These enhance resistance to enzymatic degradation and alter protein interactions [36, 60], leading to the development of potent cGMP analogues that act as competitive inhibitors by mimicking cGMP but with prolonged stability. Paquet‐Durand *et al*. [61] were the first to show that the use of cGMP analogues significantly reduced photoreceptor cell loss in organotypic retinal explants derived from rd1 or *Prph2* ^rd2^ (rd2) mice and protected photoreceptor cells in rd1 mice *in vivo*. Combined with a liposomal drug delivery system enabling retinal targeting, Vighi *et al*. [60] showed that PKG inhibition with the cGMP analogue CN03 markedly preserved morphology and retinal function in cone‐mediated electroretinogram (ERG), thus counteracting photoreceptor degeneration in rd1, rd2, and rd10 mouse models of RP. A follow‐up study using rd10 mouse retinal explants and phosphoproteomics indicated the critical role of protein kinases A, C, and G (PKA/PKC/PKG) and calcium/calmodulin‐dependent protein kinase (CAMK) in cGMP‐induced photoreceptor loss [35]. These observations suggest a crosstalk between cGMP and cAMP signaling pathways during retinal degeneration. Recent studies have refined cGMP analogues for greater specificity and stability [62, 63], while the original cGMP analogue CN03, in conjunction with advanced drug delivery systems, may be advancing toward clinical evaluation [64]. A novel PKG inhibitor CN238, as reported by Tolone *et al*. [62], demonstrated preserved photoreceptor viability and functionality in rd1 and rd10 retinal explants. However, as general cGMP inhibition can compromise cone function, a dual‐analogue approach has also been proposed to block CNG channel activity in rods while allowing continuous CNG channel activity in cones [65]. Finally, dual inhibition of CNG channel activity and PKG signaling may achieve the best effect, as the knockout of *Prkg1* was found to slow retinal degeneration in *Cngb1* ^−/−^ but not in *Pde6g* ^−/−^ mouse models [51].

To summarize, there is ample evidence supporting the therapeutic effect of cGMP pathway inhibition in PDE6 mutations. Targeting this pathway has demonstrated retinoprotective effects in experimental models also beyond the PDE6 mutation; however, more research on this topic is warranted in the search for general, mutation‐agnostic therapies for RP.

## The cAMP pathway as a drug target in retinitis pigmentosa

**Fig. 2:**
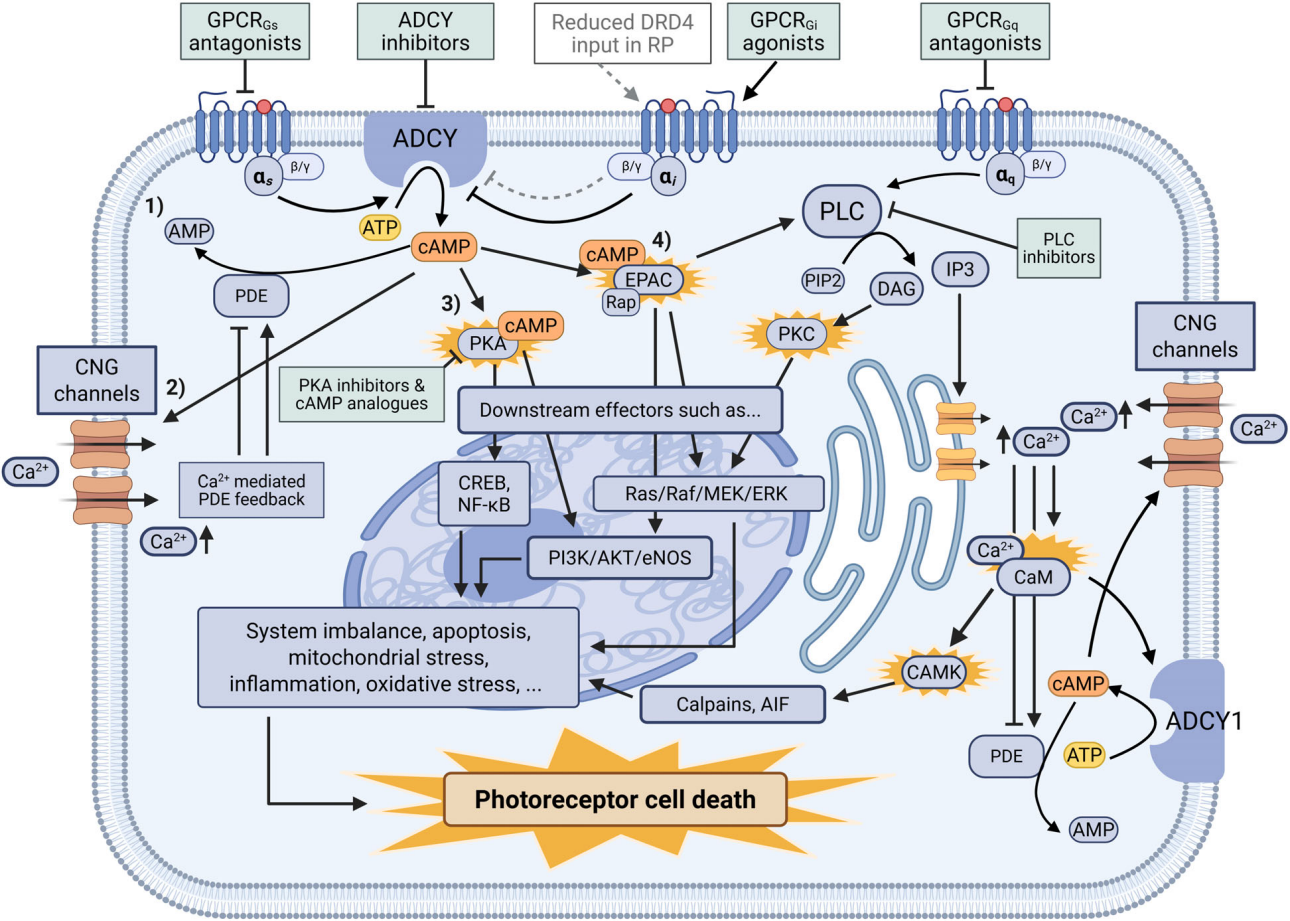
Interplay between cAMP and calcium intracellular signaling systems, with hypothetical signaling mechanisms and therapeutic targets in RP. From left to right, top to bottom: Formation of cyclic adenosine monophosphate (cAMP) occurs when adenylate cyclase (ADCY) is activated by a stimulatory G‐protein (G_s_) that has dissociated from an agonist‐induced G protein‐coupled receptor (GPCR) [66]. ADCY catalyzes the formation of cAMP from adenosine triphosphate (ATP), and this can be inhibited with the activation of a GPCR coupled with an inhibitory G‐protein (G_i_). This inhibitory effect may be diminished in RP, for instance due to mass loss of dopamine D_4_ receptors following rod degeneration [67], leaving the catecholaminergic GPCR system disbalanced. The fate of synthesized cAMP is multifactorial. It can (1) be inactivated through hydrolysis to AMP by phosphodiesterase(s) (PDE); (2) open cyclic nucleotide‐gated (CNG) ion channels to increase intracellular calcium (Ca^2+^) concentration, which in turn can modulate PDE activity in addition to altering a multitude of cellular functions; (3) activate phosphokinase A (PKA), which is often regarded as the main effector phosphorylating several target proteins [such as cAMP response element‐binding protein (CREB) and transcription factor nuclear factor kappa‐light‐chain‐enhancer of activated B cells (NF‐κB)] to relay signaling; (4) activate exchange protein activated by cAMP (EPAC), which is mainly regarded as a guanine nucleotide exchange factor for GTPase Rap and can activate phospholipase C (PLC; particularly the epsilon subtype) [66]. PLC can also be directly activated by GPCRs with a modulatory G‐protein (G_q_) coupling, and this activation leads to the degradation of phosphatidylinositol 4,5‐bisphosphate (PIP_2_) to diacylglycerol (DAG), further activating protein kinase C (PKC) and 1,4,5‐trisphosphate (IP_3_), which ultimately induces Ca^2+^ release from storages. Ca^2+^ pairing with calmodulin (CaM) drives the activation of Ca^2+^/CaM‐sensitive ADCY subtypes (such as ADCY1) to enhance cAMP generation, which in turn can increase Ca^2+^ influx *via* CNG channels. This together with the complex influence of Ca^2+^ on PDE activity creates a feedback loop between the cAMP and Ca^2+^ systems. Ca^2+^/CaM‐complex can additionally activate Ca^2+^/calmodulin‐dependent protein kinases (CAMKs), which have their own specific effects, including the activation of calpains and apoptosis‐inducing factor (AIF) [25, 66]. The possible cAMP overactivation together with dysregulated Ca^2+^ (and cGMP) system could disrupt the homeostatic downstream signaling in for instance the phosphoinositide 3‐kinase/protein kinase B/endothelial nitric oxide synthase (PI3K/AKT/eNOS) and mitogen‐activated protein kinase (Ras/Raf/MEK/ERK) pathways [66, 68]. These disruptions could exacerbate retinal degeneration in RP through different mechanisms and could be combated with drugs inhibiting cAMP/Ca^2+^ signaling. Created in BioRender. Leinonen, H. (2025) https://BioRender.com/z99r339.

The second important cyclic nucleotide and cellular signaling molecule, cAMP, is synthesized intracellularly from adenosine triphosphate (ATP) by adenylate cyclase (ADCY), which is mainly regulated by G protein‐coupled receptor (GPCR) activities [66] (Fig. 2). Various GPCRs coupled with stimulatory G_s_ or inhibitory G_i_ proteins activate or inhibit ADCYs and cAMP synthesis, respectively. cAMP can activate PKA, which regulates diverse signaling pathways by phosphorylating different target proteins and modulating ion channels, or another family of proteins called exchange proteins activated by cAMP (EPACs), which in turn stimulate small GTPases to convey downstream effects. Furthermore, this second messenger can modulate specific CNG ion channels and consequently increase inward calcium currents in the cell. In turn, calcium can influence cAMP production *via* calcium‐sensitive ADCY1 by activating calmodulin; thus, the two systems are interconnected. In the retina, cAMP localization is more diffuse throughout the tissue than cGMP, which is concentrated in the photoreceptor cilia [69]. Overall, the role and functions of cAMP in the retina are less understood than those of cGMP (reviewed in [70]). Nonetheless, cAMP plays a significant role in signaling pathways by mediating the effects of various hormones and neurotransmitters [66] and is also involved in light‐adaptive processes [71], such as photoreceptor elongation [72]. cAMP also plays a role in phototransduction, although its effect is not as crucial as that of cGMP [70]. Under dark conditions, cAMP levels are maintained at relatively high concentrations [26, 27, 69], which helps modulate the phototransduction system and aids in keeping CNG channels open during the photoreceptor ‘dark current’ process [70].

Similar to cGMP, dysregulation of cAMP signaling has been proposed to play a role in retinal degeneration [33]. Elevation of retinal cAMP levels has been demonstrated in several RP disease models, such as *Rho* ^P347S^ [26], rd2 [27], P23H, and *Rho* ^S334Ter^ rats [28]. In the context of RP, this *in vivo* cAMP increase is associated with photoreceptor death, as multiple pharmacological interventions targeting cAMP generation decelerate the progression of retinal degeneration. Receptor antagonism of various Gs‐or Gq‐linked GPCRs or agonism of different Gi‐linked GPCRs (Table 1) has been shown to enhance photoreceptor survival both morphologically and/or functionally. Examples include light‐induced retinal degeneration in albino ddY and BALB/c mice [74, 75, 80, 81, 82, 83, 84, 85], Sprague–Dawley rats [86, 87], mouse models of night blindness [73], and Stargardt disease modeling *Abca4* ^−/−^ *Rdh8* ^−/−^ mice [74, 75, 77, 78] in addition to the *Rpe65* ^−/−^ mouse model of Leber congenital amaurosis, rd10 and P23H RP mouse models, and *PDE6A* ^−/−^ dogs [67, 76]. The benefit, manifested as thicker outer nuclear layer and/or stronger ERG responses, is synergistic when multiple compounds with different GPCR targets are administered simultaneously [67, 74], revealing that multiple receptor pathways are involved.

**Table 1:** Monoaminergic GPCR targeting drugs that have been shown to mitigate retinal degeneration (RD) after acute or chronic administration in genetically modified RD models. 5‐HTR, serotonergic receptor; DR, dopaminergic receptor; P, intraperitoneal injection; SC, subcutaneous injection; αR, adrenergic alpha receptor; βR, adrenergic beta receptor; Night blindness models with full knockouts of *Arr1*, Arrestin beta 1; *Gnat1*, Guanine nucleotide‐binding protein G(t) subunit alpha‐1; *Grk1*, G Protein‐Coupled Receptor Kinase 1 [73].

Mechanism	Drugs	Paradigm	Animal model(s)	References
α1R (G_q_) antagonism	Doxazosin, prazosin, tamsulosin	IP (acute, light‐induced RD)	*Abca4* ^−/−^ *Rdh8* ^−/−^ mouse	[74]
α2R (G_i_) agonism	Lofexidine, guanabez, guanfacine	IP (acute, light‐induced RD)	*Abca4* ^−/−^ *Rdh8* ^−/−^ mouse	[74]
β1R (G_s_) antagonism	Metoprolol	IP (acute, light‐induced RD)	*Abca4* ^−/−^ *Rdh8* ^−/−^ mouse	[75]
β1/β2R (G_s_) antagonism	Metipranolol	SC/eye drops (chronic)	rd10 mouse	[76]
D2/D3R (G_i_) agonism	Bromocriptine^a^	IP (acute, light‐induced RD)	*Abca4* ^−/−^ *Rdh8* ^−/−^ mouse	[75]
5‐HT1R (G_q_) agonism	8‐OH‐DPAT	IP (acute, light‐induced RD)	Abca4^−/−^Rdh8^−/−^ mouse	[77]
5‐HT2R (G_q_) antagonism	Ketanserin, ritanserin	IP (acute, light‐induced RD)	Abca4^−/−^Rdh8^−*/−*^ mouse	[77]
5‐HT4R (G_s_) antagonism	RS23579‐190	IP (acute, light‐induced RD)	Abca4^−/−^Rdh8^−*/−*^ mouse	[74]
5‐HT6R (G_s_) antagonism	RO04‐6790, SGS518	IP (acute, light‐induced RD)	*Abca4* ^−/−^ *Rdh8* ^−/−^ mouse	[74]
5‐HT7R (G_s_) antagonism	SB269970, LY215840	IP (acute, light‐induced RD)	*Abca4* ^−/−^ *Rdh8* ^−/−^ mouse	[74]
Combined^b^ α1R and β2R antagonism and D2/D3R agonism	Tamsulosin or doxazosin/metoprolol/bromocriptine	IP (acute, light‐induced RD)	*Abca4* ^−/−^ *Rdh8* ^−/−^ mouse	[75]
	Tamsulosin/metoprolol/bromocriptine	IP (acute, light‐induced RD)	*Abca4* ^−/−^ *Rdh8* ^−/−^ mouse Arr1^−/−^, Gnat1^−/−^ and Grk1^−/−^ mouse	[73, 78]
	Tamsulosin/metoprolol/bromocriptine	Oral (chronic)/subcutaneous infusion (chronic)	rd10, P23H and *Rpe65* ^−/−^ mouse/*PDE6A* ^−/−^ dog	[67]

^a^ Bromocriptine interacts with various serotonergic and adrenergic receptors in addition to dopaminergic receptors [79].

^b^ Combining two compounds of these three has also been found beneficial, but not to the same extent [67].

Although evidence linking these treatments directly to retinal cAMP levels is absent, they hypothetically decrease cAMP production and activity by reducing ADCY activity. In line with this, the cAMP analogue 8‐bromo‐cAMP exacerbated rod cell death in a zebrafish mutant oval (ovl) model, in which a mutation in a cilium gene intraflagellar transport protein 88 homolog (*Ift88*) leads to the loss of photoreceptor outer segments [88]. Additionally, in the same study, Nakao *et al*. showed that inhibition of PKA, the downstream effector of cAMP, using KT5720 provided rod protection in ovl zebrafish. The detrimental role of elevated cAMP is further supported by the finding that inhibition of the ADCY enzyme with SQ22536 decelerates retinal degeneration by improving rod cell survival and outer nuclear layer morphology in ovl zebrafish, rd10 [88] and *Abca4* ^−/−^ *Rdh8* ^−/−^ mice [74]. Moreover, the retinal proteomic expressions of different ADCY subtypes are upregulated in P23H (ADCY2, ADCY5, and ADCY8) and rd10 mice (ADCY2) [33].

However, unlike cGMP, the causal link between cAMP dysregulation and retinal degeneration progression/severity has not been systematically demonstrated, and the effects of abnormal cAMP levels are likely to be paradigm‐, disease‐, and cell‐specific. For instance, the elevation of cAMP by forskolin, an ADCY activator, can improve the survival of retinal ganglion cells *in vitro* [89, 90], and specific PDE inhibitors, which should enhance cyclic nucleotide signaling, have been shown to mitigate retinal degeneration in rd10 mice [91].

### Does the loss of dopamine D4 receptor input influence cAMP system dysregulation?

One major known contributor affecting cAMP levels in the retina is the circadian regulated dopamine‐melatonin feedback loop, in which light‐induced dopamine release inhibits cAMP synthesis *via* dopamine D4 receptor (DRD4) activity, whereas melatonin increases cAMP by inhibiting dopamine release [92, 93]. Thus, the retinal cAMP concentration is lower in the light‐adapted state than in the dark‐adapted state [26, 27, 69].

DRD4's role in the regulation of photoreceptor physiology and adaptation to light, as well as in maintaining the excitation‐inhibition balance in retinal signaling, is well‐known (comprehensively reviewed [94]). DRD4 is highly expressed in rod photoreceptor cells [95, 96], the primary degeneration locus in RP. Decreasing rod cell count likely explains the consequent decrease in retinal *Drd4* expression in rd10 mice [67], which could hypothetically lead to system dysregulation *via* decreased inhibitory input on cAMP synthesis. Genetic *Ddr4* ^−/−^ knockout and pharmacological D4‐antagonism by L‐745870 decreased the expression of retinal ADCY1 in mice [97]. Thus, although the downregulation of ADCY1 in RP has not been reported, the upregulation of other ADCY subtypes in the retinas of RP mice [33] could be an adaptive outcome. Furthermore, in *Ddr4* ^−/−^ mouse retinas, cAMP levels have been reported to increase *in vitro* when exposed to light, and this elevation can be reversed with an antagonist of the G_s_‐coupled dopamine D1 receptor [71], indicating that the roles of other dopamine receptors may become more significant in the absence of DRD4. This could potentially be applied to other GPCRs outside the dopaminergic family, such as adrenergic or serotonergic receptors, as suggested by Table 1, which could consequently lead to further dysregulation in cAMP‐related cellular dynamics.

### The complex interaction between G protein‐coupled receptors, cAMP, and intracellular calcium

cAMP signaling closely interacts with systems that regulate intracellular calcium [66], which is highly relevant in the context of RP. In addition to the therapeutic effects of calcium channel inhibitors (as briefly discussed above), the inhibition of intracellular calcium signaling afforded by certain GPCR drugs, such as the adrenergic alpha‐1 antagonist tamsulosin (Table 1), shows therapeutic potential in retinal degeneration. Receptor agonism of GPCRs coupled with G_q_ protein causes the activation of phospholipase C (PLC), which in turn leads to the generation of additional second messengers, inositol 1,4,5‐trisphosphate (IP3) and diacylglycerol (DAG) [66] (Fig. 2). DAG can activate PKC, whereas IP3 increases the amount of available calcium. Calcium can, for instance, bind to calmodulin to activate CAMKs and the calmodulin‐sensitive ADCY1 subtype, which further convey various downstream effects to modulate, for example, gene transcription and the cAMP system. Inhibition of PLC and downstream calcium release has been shown to alleviate light‐induced retinal damage [77]. Furthermore, multiple CAMK subtypes are upregulated by retinal degeneration at the protein level in the retinas of P23H and rd10 mice [33]. Lack of DRD4 expression has been shown to modulate calmodulin‐dependent ADCY1 activity in addition to reducing its expression [97]. Nir *et al*. suggested that in the absence of retinal DRD4, the system adapts by regulating the expression of compounds involved in the cascades [71], which could explain the proteomic upregulation of both CAMKs and ADCYs in RP *in vivo* [33]. Furthermore, this regulation might involve changes in the additional cAMP effector EPAC, as inhibition of EPAC1 and the consequent activation of CAMKII could be neuroprotective as indicated by results from a mouse ocular hypertension model [98], and EPAC2 expression shows upregulation in P23H RP mice [33]. *Epac1* deletion has additionally been shown to decrease pathological angiogenesis in a mouse model of oxygen‐induced retinopathy, which also presents with elevated retinal cAMP levels [68]. More research on this complex, cell‐specific system and its downstream effects (Fig. 2) is warranted for further mechanistic understanding in the context of retinal degeneration.

## Drug repurposing aspects

Drug repurposing, that is, the use of existing drug compounds for a condition they were not originally developed to treat, could provide a widely applicable therapy faster than the extensive application of targeted gene therapies in RP. Although both cyclic nucleotide pathways are potential targets for drug repurposing, cAMP may be more readily applicable because of the large number of existing drugs, particularly GPCR drugs, that affect this system. To the best of our knowledge, the cGMP synthesis suppressor MMF is the only cGMP system‐targeting drug that is under investigation for retinal degeneration and has already been approved for clinical use for other indications. MMF is an immunosuppressor for organ transplantation patients and is commonly used off‐label as an immunomodulatory medication for the treatment of ocular inflammation [99, 100].

In contrast, targeting cAMP signaling with GPCR drugs in chronic RP models has demonstrated the potential of multiple compounds that have already been approved for clinical use for other indications. The selective β1R antagonist metoprolol and the unselective β1/β2R antagonist metipranolol are primarily prescribed to treat cardiovascular disease and glaucoma, respectively [76, 101]. Furthermore, adrenergic α1R antagonists, such as doxazosin, prazosin, and tamsulosin, are commonly used to treat symptoms of benign prostatic hyperplasia [102], whereas inhibitory dopaminergic drugs such as pramipexole and bromocriptine are both used in Parkinson's disease [79]. Importantly, combination treatment with tamsulosin, metoprolol, and bromocriptine has proven to be beneficial across multiple disease paradigms with lower doses than what would be needed for the same effect with individual drugs [67, 74].

## Drug delivery aspects

While the drugs listed above generally display favorable safety profiles, direct targeting of retinal cAMP or cGMP signaling, with, for example, ADCY/GUCY inhibitors or cyclic nucleotide analogues, should ideally not be orally administered due to the ubiquitous expression of these targets in the body. Even when targeting GPCRs, systemic drug treatment may lead to an unacceptable degree of side effects with the therapeutic doses needed to significantly mitigate retinal degeneration; thus, the development of drug delivery systems for ocular targeting is justified. Retinal drug delivery is challenging because of anatomical and physiological barriers, including the corneal barrier, tear fluid turnover, blood–retinal barrier (BRB), and scleral/chorioretinal layers, which limit the efficacy of retinal therapeutics (see e.g., [103, 104], for reviews). Topical or systemic administration, although preferred for patient compliance and long‐term use, often achieves insufficient retinal bioavailability, particularly for biologics. Consequently, intraocular drug delivery *via* intravitreal injection (IVI) remains the standard clinical delivery method for major retinal diseases leading to vision loss. However, IVI is invasive, necessitates frequent dosing (may require intervals of 4 weeks), and poses risks such as endophthalmitis, retinal detachment, and patient discomfort. Additionally, IVI is often unsuitable for gene therapy and other therapies that require intracellular delivery, particularly in the outer retinal compartments.

Topical eye drops, while patient‐friendly, face rapid tear clearance and poor retinal penetration, with less than 5% of the instilled dose reaching the posterior segments of the eye [105]. Despite these challenges, topical eye drops of potent small molecules (e.g., dexamethasone and dorzolamide) have shown efficacy in posterior segment diseases. Emerging strategies to enhance topical delivery are crucial for ensuring adequate retinal exposure and include prodrug formulations, mucoadhesive gels, viscosity, and permeability enhancers to mitigate washout and corneal barriers (reviewed in [106]). In clinical trials of age‐related macular degeneration, a topical regorafenib formulation that inhibits vascular endothelial growth factor (VEGF) kinases failed in a phase 2 trial as it was terminated due to insufficient efficacy and retinal drug exposure [107], while an anti‐VEGF PAN‐90806 eye drop agent demonstrated promising efficacy in clinical trials after 12 weeks [108, 109]. These results highlight the challenge of developing a universally reproducible formulation for topical drug delivery to the retina.

Systemic drug delivery *via* oral or intravenous routes is constrained by the blood–retinal barrier (BRB), often requiring high doses that increase the risk of systemic toxicity. Nevertheless, systemic agents such as oral carbonic anhydrase inhibitors and intravenous corticosteroids remain clinically relevant for infectious or inflammatory conditions of the eye (e.g., retinitis, macular edema, or uveitis) [110]. In many cases, systemic drug delivery may need to be coupled with targeted delivery approaches to enable the effective treatment of chronic retinal diseases [111]. Innovative device‐assisted and nanotechnology‐based approaches aim to overcome the off‐target toxicity limitations associated with systemic delivery [112]. For instance, transscleral iontophoresis enhances the delivery of biologics (e.g., bevacizumab) in preclinical studies [113]. Moreover, focused ultrasound combined with microbubbles temporarily disrupts the BRB, facilitating drug entry from circulation [114]. Finally, nanocarriers, including liposomes, extracellular vesicles, dendrimers, and polymeric nanoparticles, improve ocular retention, enable tissue‐specific delivery, and reduce dosing frequency, potentially providing an alternative for conventional IVI delivery in the future [115].

## Concluding remarks and future directions

Investigation of cGMP targeting as a therapeutic approach for RP shows promise. The premise of targeting the cGMP pathway is rooted in its critical role in phototransduction and regulation of CNG ion channels, which are essential for normal visual function. Dysregulation of cGMP levels, particularly the pathological accumulation of cGMP, has been causally linked to retinal degeneration caused by PDE6 defects. The development of specific cGMP analogues and inhibitors, along with gene therapy approaches, offers promising avenues for therapeutic intervention. Future research should continue to explore optimization of cGMP targeting to maintain basal visual function, as well as to improve ocular drug targeting.

Exploration of cAMP targeting as a therapeutic strategy for retinal degeneration, particularly RP, is also attractive. The potential of cAMP system targeting lies in its role as a crucial cellular signaling pathway, which, when dysregulated, is implicated in the progression of retinal degeneration. Elevated cAMP levels have been observed in various retinal degeneration models, and pharmacological interventions that reduce cAMP synthesis or activity have shown potential to decelerate the disease course. Drug repurposing strategies using existing drugs that modulate the cAMP system offer a faster route to potential therapies than the development of new targeted treatments. However, cAMP targeting poses several challenges. Foremost, the causal link between cAMP dysregulation and retinal degeneration has not been systematically demonstrated, and the effects of abnormal cAMP levels may vary depending on the disease paradigm and the cell type. Therefore, although several *in vivo* studies are promising, translating these findings into clinical practice involves overcoming many unresolved questions. The safety and efficacy of repurposed drugs for long‐term use in patients with retinal degeneration need to be tested in clinical trials, and off‐label use is discouraged. Nevertheless, we propose the cGMP and cAMP systems as promising therapeutic targets to alleviate RP.

## Conflict of interest

The authors declare no conflict of interest.

## Author contributions

KV, ABM, and HL wrote the initial draft of the manuscript. KV, ABM, and HL revised and edited the manuscript.

## References

[1] Hartong D , Berson E and Dryja T (2006) Retinitis pigmentosa. Lancet 368, 1795–1809. 17113430 10.1016/S0140-6736(06)69740-7

[2] Ayuso C and Millan J (2010) Retinitis pigmentosa and allied conditions today: a paradigm of translational research. Genome Med 2, 34. 20519033 10.1186/gm155 PMC2887078

[3] Vaidya P and Vaidya A (2015) Ophthalmology and clinical research retinitis pigmentosa: disease encumbrance in the eurozone. Int J Ophthalmol Clin Res 2, 30.

[4] RetNet.org Summaries of Genes and Loci Causing Retinal Diseases. Accessed 22.4.2025.

[5] Heath JR , Mukhtar S , McAllister I , Morgan WH , Mackey DA and Chen FK (2021) Inherited retinal diseases are the most common cause of blindness in the working‐age population in Australia. Ophthalmic Genet 42, 431–439. 33939573 10.1080/13816810.2021.1913610 PMC8315212

[6] Luxturna U.S. Food and Drug Administration. Accessed 25.4.2025. https://www.fda.gov.

[7] Safety and Tolerability of Intravitreal Administration of VG901 in Patients With Retinitis Pigmentosa Due to Mutations in the CNGA1 Gene. Clinicaltrials.gov, NCT06291935.

[8] Promising ROd‐cone DYstrophy Gene TherapY (PRODYGY). Clinicaltrials.gov, NCT05748873.

[9] Safety and Efficacy Study in Patients With Retinitis Pigmentosa Due to Mutations in PDE6B Gene. Clinicaltrials.gov, NCT03328130.

[10] A First‐in‐human, Proof of Concept Study of CPK850 in Patients With RLBP1 Retinitis Pigmentosa. Clinicaltrials.gov, NCT03374657.

[11] A Phase 3 Study Of OCU400 Gene Therapy for the Treatment Of Retinitis Pigmentosa (liMeliGhT). Clinicaltrials.gov, NCT06388200.

[12] A Study to Evaluate the Safety and Tolerability of QR‐1123 in Subjects With Autosomal Dominant Retinitis Pigmentosa Due to the P23H Mutation in the RHO Gene (AURORA). Clinicaltrials.gov, NCT04123626.

[13] 4D‐125 in Patients With X‐Linked Retinitis Pigmentosa (XLRP). Clinicaltrials.gov, NCT04517149.

[14] Gene Therapy for RPGR Gene Mutation‐associated X‐linked Retinitis Pigmentosa. Clinicaltrials.gov, NCT06492850.

[15] Follow‐up Gene Therapy Trial for the Treatment of X‐linked Retinitis Pigmentosa Associated With Variants in the RPGR Gene. Clinicaltrials.gov, NCT04794101.

[16] Gene Therapy for Subjects With RPGR Mutation‐associated X‐linked Retinitis Pigmentosa. Clinicaltrials.gov, NCT05874310.

[17] A Study Comparing Two Doses of AGTC‐501 in Male Participants With X‐linked Retinitis Pigmentosa Caused by RPGR Mutations (DAWN). Clinicaltrials.gov, NCT06275620.

[18] Long‐term Follow‐up Gene Therapy Study for RPGR‐XLRP. Clinicaltrials.gov, NCT04312672.

[19] A Study Comparing Two Doses of AGTC‐501 in Male Subjects With X‐linked Retinitis Pigmentosa Caused by RPGR Mutations (SKYLINE). Clinicaltrials.gov, NCT06333249.

[20] A Follow‐on Study for Second‐Eye Treatment for Participants Previously Treated With Gene Therapy for X‐Linked Retinitis Pigmentosa (XLRP). Clinicaltrials.gov, NCT06646289.

[21] Safety and Efficacy of rAAV2tYF‐GRK1‐RPGR in Subjects With X‐linked Retinitis Pigmentosa Caused by RPGR Mutations (HORIZON). Clinicaltrials.gov, NCT03316560.

[22] A Clinical Trial Evaluating the Safety and Efficacy of a Single Subretinal Injection of AGTC‐501 in Participants With XLRP. Clinicaltrials.gov, NCT04850118.

[23] Study to Evaluate Ultevursen in Subjects With Retinitis Pigmentosa (RP) Due to Mutations in Exon 13 of the USH2A Gene (LUNA). Clinicaltrials.gov, NCT06627179.

[24] Schmidt K‐G , Bergert H and Funk RHW (2008) Neurodegenerative diseases of the retina and potential for protection and recovery. Curr Neuropharmacol 6, 164–178. 19305795 10.2174/157015908784533851 PMC2647152

[25] Newton F and Megaw R (2020) Mechanisms of photoreceptor death in retinitis pigmentosa. Genes 11, 1120. 32987769 10.3390/genes11101120 PMC7598671

[26] Weiss E , Hao Y , Dickerson C , Weiss ER , Dickerson CD , Osawa S , Shi W , Zhang L and Wong F (1995) Altered cAMP levels in retinas from transgenic mice expressing a rhodopsin mutant. Biochem Biophys Res Commun 216, 755–761. 7488190 10.1006/bbrc.1995.2686

[27] Nir I , Haque R and Iuvone PM (2001) Regulation of cAMP by light and dopamine receptors is dysfunctional in photoreceptors of dystrophic retinal degeneration slow (rds) mice. Exp Eye Res 73, 265–272. 11446777 10.1006/exer.2001.1037

[28] Traverso V , Bush RA , Sieving PA and Deretic D (2002) Retinal cAMP levels during the progression of retinal degeneration in rhodopsin P23H and S334ter transgenic rats. Invest Ophthalmol Vis Sci 43, 1655–1661. 11980887

[29] Chen Y and Palczewski K (2016) Systems pharmacology links GPCRs with retinal degenerative disorders. Annu Rev Pharmacol Toxicol 56, 273–298. 25839098 10.1146/annurev-pharmtox-010715-103033 PMC4580525

[30] Biasi A , Marino V , Dal Cortivo G , Maltese PE , Modarelli AM , Bertelli M , Colombo L and Dell'Orco D (2021) A novel guca1a variant associated with cone dystrophy alters cgmp signaling in photoreceptors by strongly interacting with and hyperactivating retinal guanylate cyclase. Int J Mol Sci 22, 10809. 34639157 10.3390/ijms221910809 PMC8509414

[31] Kandaswamy S , Zobel L , John B , Santhiya ST , Bogedein J , Przemeck GKH , Gailus‐Durner V , Fuchs H , Biel M , de Angelis MH *et al*. (2022) Mutations within the cGMP‐binding domain of CNGA1 causing autosomal recessive retinitis pigmentosa in human and animal model. Cell Death Dis 8, 387. 10.1038/s41420-022-01185-0 PMC9482621 36115851

[32] Occelli LM , Zobel L , Stoddard J , Wagner J , Pasmanter N , Querubin J , Renner LM , Reynaga R , Winkler PA , Sun K *et al*. (2023) Development of a translatable gene augmentation therapy for CNGB1‐retinitis pigmentosa. Mol Ther 31, 2028–2041. 37056049 10.1016/j.ymthe.2023.04.005 PMC10362398

[33] Montaser AB , Gao F , Peters D , Vainionpää K , Zhibin N , Skowronska‐Krawczyk D , Figeys D , Palczewski K and Leinonen H (2024) Retinal proteome profiling of inherited retinal degeneration across three different mouse models suggests common drug targets in retinitis pigmentosa. Mol Cell Proteomics 23, 100855. 39389360 10.1016/j.mcpro.2024.100855 PMC11602984

[34] Fu Y and Yau KW (2007) Phototransduction in mouse rods and cones. Pflugers Arch 454, 805–819. 17226052 10.1007/s00424-006-0194-y PMC2877390

[35] Roy A , Zhou J , Nolet M , Welinder C , Zhu Y , Paquet‐Durand F , Groten J , Tomar T and Ekström P (2024) Integrative kinase activity profiling and Phosphoproteomics of rd10 mouse retina during cGMP‐dependent retinal degeneration. Int J Mol Sci 25, 3446. 38542418 10.3390/ijms25063446 PMC10970885

[36] Tolone A , Belhadj S , Rentsch A , Schwede F and Paquet‐Durand F (2019) The cGMP pathway and inherited photoreceptor degeneration: targets, compounds, and biomarkers. Genes 10, 453. 31207907 10.3390/genes10060453 PMC6627777

[37] Marmor MF (1990) Control of subretinal fluid: experimental and clinical studies. Eye 4, 340–344. 2199242 10.1038/eye.1990.46

[38] Diederen R , la Heij E , Markerink‐Van Ittersum M , Kijlstra A , Hendrikse F and de Vente J (2007) Cyclic GMP synthesis by human retinal pigment epithelial cells is mainly mediated via the particulate guanylyl cyclase pathway. Ophthalmic Res 39, 55–59. 17179739 10.1159/000098015

[39] Power M , Das S , Schütze K , Marigo V , Ekström P and Paquet‐Durand F (2020) Cellular mechanisms of hereditary photoreceptor degeneration – focus on cGMP. Prog Retin Eye Res 74, 100772. 31374251 10.1016/j.preteyeres.2019.07.005

[40] Sothilingam V , Garrido MG , Jiao K , Garcia Garrido M , Buena‐Atienza E , Sahaboglu A , Trifunović D , Balendran S , Koepfli T , Mühlfriedel R *et al*. (2015) Retinitis pigmentosa: impact of different Pde6a point mutations on the disease phenotype. Hum Mol Genet 24, 5486–5499. 26188004 10.1093/hmg/ddv275

[41] Li S , Ma H , Yang F and Ding X (2023) cGMP signaling in photoreceptor degeneration. Int J Mol Sci 24, 11200. 37446378 10.3390/ijms241311200 PMC10342299

[42] Tosi J , Davis R , Wang NK , Davis RJ , Naumann M , Lin C‐S and Tsang SH (2011) shRNA knockdown of guanylate cyclase 2eorcyclic nucleotide gated channel alpha 1increases photoreceptor survival in a cGMP phosphodiesterase mouse model of retinitis pigmentosa. J Cell Mol Med 15, 1778–1787. 20950332 10.1111/j.1582-4934.2010.01201.x PMC3071858

[43] Sahaboglu A , Tanimoto N , Kaur J , Sancho‐Pelluz J , Huber G , Fahl E , Arango‐Gonzalez B , Zrenner E , Ekström P , Löwenheim H *et al*. (2010) PARP1 gene knock‐out increases resistance to retinal degeneration without affecting retinal function. PLoS One 5, e15495. 21124852 10.1371/journal.pone.0015495 PMC2990765

[44] Yang P , Lockard R , Titus H , Hiblar J , Weller K , Wafai D , Weleber RG , Duvoisin RM , Morgans CW and Pennesi ME (2020) Suppression of cGMP‐dependent photoreceptor cytotoxicity with mycophenolate is neuroprotective in murine models of retinitis pigmentosa. Invest Ophthalmol Vis Sci 61, 25. 10.1167/iovs.61.10.25 PMC7441375 32785677

[45] Zhang J , Richmond A and Ogilvie J (2014) Inhibition of dopamine signaling suppresses cGMP accumulation in rd1 retinal organ cultures. Neuroreport 25, 601–606. 24614363 10.1097/WNR.0000000000000145 PMC4275426

[46] Wu Y , Wan X , Zhao D , Chen X , Wang Y , Tang X , Li J , Li S , Sun X , Bi C *et al*. (2023) AAV‐mediated base‐editing therapy ameliorates the disease phenotypes in a mouse model of retinitis pigmentosa. Nat Commun 14, 4923. 37582961 10.1038/s41467-023-40655-6 PMC10427680

[47] Frasson M , Sahel J , Fabre M , Sahel JA , Simonutti M , Dreyfus H and Picaud S (1999) Retinitis pigmentosa: rod photoreceptor rescue by a calcium‐channel blocker in the rd mouse. Nat Med 5, 1183–1187. 10502823 10.1038/13508

[48] Takano Y , Ohguro H , Dezawa M , Ishikawa H , Yamazaki H , Ohguro I , Mamiya K , Metoki T , Ishikawa F and Nakazawa M (2004) Study of drug effects of calcium channel blockers on retinal degeneration of rd mouse. Biochem Biophys Res Commun 313, 1015–1022. 14706644 10.1016/j.bbrc.2003.12.034

[49] Nakazawa M , Ohguro H , Takeuchi K , Miyagawa Y , Ito T and Metoki T (2011) Effect of nilvadipine on central visual field in retinitis pigmentosa: a 30‐month clinical trial. Ophthalmologica 225, 120–126. 20948238 10.1159/000320500

[50] Nakazawa M , Suzuki Y , Ito T , Metoki T , Kudo T and Ohguro H (2013) Long‐term effects of nilvadipine against progression of the central visual field defect in retinitis pigmentosa: an extended study. Biomed Res Int 2013, 585729. 24319686 10.1155/2013/585729 PMC3844269

[51] Wang T , Tsang S and Chen J (2017) Two pathways of rod photoreceptor cell death induced by elevated cGMP. Hum Mol Genet 26, 2299–2306. 28379353 10.1093/hmg/ddx121 PMC6075581

[52] Wang T , Reingruber J , Woodruff M , Woodruff ML , Majumder A , Camarena A , Artemyev NO , Fain GL and Chen J (2018) The PDE6 mutation in the rd10 retinal degeneration mouse model causes protein mislocalization and instability and promotes cell death through increased ion influx. J Biol Chem 293, 15332–15346. 30126843 10.1074/jbc.RA118.004459 PMC6177582

[53] Barabas P , Peck CC and Krizaj D (2010) Do calcium channel blockers rescue dying photoreceptors in the pde6b rd1 mouse? Adv Exp Med Biol 664, 491–499. 20238051 10.1007/978-1-4419-1399-9_56 PMC2921874

[54] Pearce‐Kelling S , Aleman T , Nickle A , Laties AM , Aguirre GD , Jacobson SG and Acland GM (2001) Calcium channel blocker D‐cis‐diltiazem does not slow retinal degeneration in the PDE6B mutant rcd1 canine model of retinitis pigmentosa. Mol Vis 25, 42–47. 11239245

[55] Roy A , Tolone A , Hilhorst R , Groten J , Tomar T and Paquet‐Durand F (2022) Kinase activity profiling identifies putative downstream targets of cGMP/PKG signaling in inherited retinal neurodegeneration. Cell Death Dis 8, 93. 10.1038/s41420-022-00897-7 PMC8894370 35241647

[56] Hofmann F , Bernhard D , Lukowski R and Weinmeister P (2009) cGMP regulated protein kinases (cGK). cGMP: generators, effectors and therapeutic implications. Handb Exp Pharmacol 191, 137–191. 10.1007/978-3-540-68964-5_8 19089329

[57] Ekström P , Ueffing M , Zrenner E and Paquet‐Durand F (2014) Novel in situ activity assays for the quantitative molecular analysis of neurodegenerative processes in the retina. Curr Med Chem 21, 3478–3493. 24934347 10.2174/0929867321666140601201337

[58] Gamm D , Barthel L , Raymond P and Uhler M (2000) Localization of cGMP‐dependent protein kinase isoforms in mouse eye. Invest Ophthalmol Vis Sci 41, 2766–2773. 10937596

[59] Lee J , Li S , Hsu S , Lee JH , Liu T , Kim C , Woods VL and Casteel DE (2011) The amino terminus of cGMP‐dependent protein kinase Iβ increases the dynamics of the protein's cGMP‐binding pockets. Int J Mass Spectrom 302, 44–52. 21643460 10.1016/j.ijms.2010.07.021 PMC3107041

[60] Vighi E , Trifunovic D , Veiga‐Crespo P , Rentsch A , Hoffmann D , Sahaboglu A , Strasser T , Kulkarni M , Bertolotti E , van den Heuvel A *et al*. (2018) Combination of cGMP analogue and drug delivery system provides functional protection in hereditary retinal degeneration. Proc Natl Acad Sci U S A 115, E2997–E3006. 29531030 10.1073/pnas.1718792115 PMC5879685

[61] Paquet‐Durand F , Hauck SM , van Veen T , Ueffing M and Ekström P (2009) PKG activity causes photoreceptor cell death in two retinitis pigmentosa models. J Neurochem 108, 796–810. 19187097 10.1111/j.1471-4159.2008.05822.x

[62] Tolone A , Haq W , Fachinger A , Roy A , Kesh S , Rentsch A , Wucherpfennig S , Zhu Y , Groten J , Schwede F *et al*. (2023) The PKG inhibitor CN238 affords functional protection of photoreceptors and ganglion cells against retinal degeneration. Int J Mol Sci 24, 15277. 37894958 10.3390/ijms242015277 PMC10607377

[63] Pérez O , Stanzani A , Huang L , Schipper N , Loftsson T , Bollmark M and Marigo V (2024) New improved cGMP analogues to target rod photoreceptor degeneration. J Med Chem 67, 8396–8405. 38688030 10.1021/acs.jmedchem.4c00586 PMC11129186

[64] EraLearn Translating cGMP analogues into a treatment for retinitis pigmentosa, EJPRD20‐101. 2nd EJP RD Joint Transnational Call for Rare Diseases Research Project (JTC 2020). https://www.era‐learn.eu, accessed 29.4.2025.

[65] Wucherpfennig S , Haq W , Popp V , Kesh S , Das S , Melle C , Rentsch A , Schwede F , Paquet‐Durand F and Nache V (2022) cGMP analogues with opposing actions on CNG channels selectively modulate rod or cone photoreceptor function. Pharmaceutics 14, 2102. 36297537 10.3390/pharmaceutics14102102 PMC9612005

[66] Sassone‐Corsi P (2012) The cyclic AMP pathway. Cold Spring Harb Perspect Biol 4, a011148. 23209152 10.1101/cshperspect.a011148 PMC3504441

[67] Leinonen H , Zhang J , Occelli LM , Seemab U , Choi EH , L.P. Marinho LF , Querubin J , Kolesnikov AV , Galinska A , Kordecka K *et al*. (2024) A combination treatment based on drug repurposing demonstrates mutation‐agnostic efficacy in pre‐clinical retinopathy models. Nat Commun 15, 5943. 39009597 10.1038/s41467-024-50033-5 PMC11251169

[68] Liu H , Mei F , Yang W , Wang H , Wong E , Cai J *et al*. (2020) Epac1 inhibition ameliorates pathological angiogenesis through coordinated activation of notch and suppression of VEGF signaling. Sci Adv 6, eaay3466. 10.1126/sciadv.aay3566 PMC6938696 31911948

[69] Orr H , Lowry O , Cohen A and Ferrendelli J (1976) Distribution of 3′:5′‐cyclic AMP and 3′:5′‐cyclic GMP in rabbit retina in vivo: selective effects of dark and light adaptation and ischemia. Proc Natl Acad Sci U S A 73, 4442–4445. 188039 10.1073/pnas.73.12.4442 PMC431491

[70] Erofeeva N , Meshalkina D and Firsov M (2023) Multiple roles of cAMP in vertebrate retina. Cells 12, 1157. 37190066 10.3390/cells12081157 PMC10136742

[71] Nir I , Harrison J , Haque R , Harrison JM , Low MJ , Grandy DK , Rubinstein M and Iuvone PM (2002) Dysfunctional light‐evoked regulation of cAMP in photoreceptors and abnormal retinal adaptation in mice lacking dopamine D4 receptors. J Neurosci 22, 2063–2073. 11896146 10.1523/JNEUROSCI.22-06-02063.2002 PMC6758276

[72] Stenkamp D , Iuvone P and Adler R (1994) Photomechanical movements of cultured embryonic photoreceptors: regulation by exogenous neuromodulators and by a regulable source of endogenous dopamine. J Neurosci 14, 3083–3096. 7910204 10.1523/JNEUROSCI.14-05-03083.1994 PMC6577438

[73] Leinonen H , Choi EH , Gardella A , Kefalov VJ and Palczewski K (2019) A mixture of U.S. food and drug administration‐approved monoaminergic drugs protects the retina from light damage in diverse models of night blindness. Invest Ophthalmol Vis Sci 60, 1442–1453. 30947334 10.1167/iovs.19-26560 PMC6736410

[74] Chen Y , Palczewska G , Mustafi D , Golczak M , Dong Z , Sawada O , Maeda T , Maeda A and Palczewski K (2013) Systems pharmacology identifies drug targets for Stargardt disease‐associated retinal degeneration. J Clin Invest 123, 5119–5134. 24231350 10.1172/JCI69076 PMC3859412

[75] Chen Y , Palczewska G , Masuho I , Gao S , Jin H , Dong Z , Gieser L , Brooks MJ , Kiser PD , Kern TS *et al*. (2016) Synergistically acting agonists and antagonists of G protein‐coupled receptors prevent photoreceptor cell degeneration. Sci Signal 9, ra74. 27460988 10.1126/scisignal.aag0245 PMC4972460

[76] Kanan Y , Khan M , Lorenc V , Da Long RC , Sciamanna J , Green K and Campochiaro PA (2019) Metipranolol promotes structure and function of retinal photoreceptors in the rd10 mouse model of human retinitis pigmentosa. J Neurochem 148, 307–318. 30315650 10.1111/jnc.14613 PMC12554146

[77] Chen Y , Okano K , Maeda T , Chauhan V , Golczak M , Maeda A and Palczewski K (2012) Mechanism of all‐trans‐retinal toxicity with implications for stargardt disease and age‐related macular degeneration. J Biol Chem 287, 5059–5069. 22184108 10.1074/jbc.M111.315432 PMC3281612

[78] Orban T , Leinonen H , Getter T , Dong Z , Sun W , Gao S , Veenstra A , Heidari‐Torkabadi H , Kern TS , Kiser PD *et al*. (2018) A combination of G protein‐coupled receptor modulators protects photoreceptors from degeneration. J Pharmacol Exp Ther 364, 207–220. 29162627 10.1124/jpet.117.245167 PMC5771314

[79] Millan M , Maiofiss L , Cussac D , Millan MJ , Audinot V , Boutin JA and Newman‐Tancredi A (2002) Differential actions of antiparkinson agents at multiple classes of monoaminergic receptor. I. A multivariate analysis of the binding profiles of 14 drugs at 21 native and cloned human receptor subtypes. J Pharmacol Exp Ther 303, 791–804. 12388666 10.1124/jpet.102.039867

[80] Shibagaki K , Okamoto K , Katsuta O and Nakamura M (2015) Beneficial protective effect of pramipexole on light‐induced retinal damage in mice. Exp Eye Res 139, 64–72. 26213307 10.1016/j.exer.2015.07.007

[81] Tullis B , Ryals R , Coyner A , Tullis BE , Ryals RC , Coyner AS , Gale MJ , Nicholson A , Ku C , Regis D *et al*. (2015) Sarpogrelate, a 5‐HT2A receptor antagonist, protects the retina from light‐induced retinopathy. Invest Ophthalmol Vis Sci 56, 4560–4569. 26200496 10.1167/iovs.15-16378 PMC4515947

[82] Coyner A , Ryals R , Ku C , Coyner AS , Ryals RC , Ku CA , Fischer CM , Patel RC , Datta S , Yang P *et al*. (2016) Retinal neuroprotective effects of flibanserin, an FDA‐approved dual serotonin receptor agonist‐antagonist. PLoS One 11, e0159776. 27447833 10.1371/journal.pone.0159776 PMC4957778

[83] Ku CA , Ryals RC , Jiang D , Coyner AS , Weller KK , Sinha W , Robb BM , Yang P and Pennesi ME (2018) The role of ERK1/2 activation in sarpogrelate‐mediated neuroprotection. Invest Ophthalmol Vis Sci 59, 462–471. 29368005 10.1167/iovs.17-23159 PMC5786286

[84] Tanaka M , Inoue Y , Yoshino Y , Kuse Y , Tanida N , Takahashi K , Miyamoto Y and Hara H (2021) Ropinirole prevents light‐induced retinal photoreceptor damage in mice. BPB Reports 4, 1–5.

[85] Pazur E , Kalatanova A , Tasker NR , Pazur EJ , Vainionpää K , Leinonen H and Wipf P (2024) Synthesis and biological analysis of iso‐dimethyltryptamines in a model of light‐induced retinal degeneration. ACS Med Chem Lett 15, 1049–1056. 39015263 10.1021/acsmedchemlett.4c00130 PMC11247652

[86] Sugawara T , Sieving PA , Michael Iuvone P and Bush RA (1998) The melatonin antagonist Luzindole protects retinal photoreceptors from light damage in the rat. Invest Ophthalmol Vis Sci 39, 2458–2465. 9804154

[87] Collier R , Patel Y , Martin E , Collier RJ , Martin EA , Dembinska O , Hellberg M , Krueger DS , Kapin MA and Romano C (2011) Agonists at the serotonin receptor (5‐HT1A) protect the retina from severe photo‐oxidative stress. Invest Ophthalmol Vis Sci 52, 2118–2126. 21087971 10.1167/iovs.10-6304

[88] Nakao T , Tsujikawa M , Notomi S , Ikeda Y and Nishida K (2012) The role of mislocalized phototransduction in photoreceptor cell death of retinitis pigmentosa. PLoS One 7, e32472. 22485131 10.1371/journal.pone.0032472 PMC3317642

[89] Meyer‐Franke A , Kaplan MR , Pfrieger FW and Barres BA (1995) Characterization of the signaling interactions that promote the survival and growth of developing retinal ganglion cells in culture. Neuron 15, 805–819. 7576630 10.1016/0896-6273(95)90172-8

[90] Shim M , Kim K , Bu J , Nam HS , Jeong SW , Park TL , Ellisman MH , Weinreb RN and Ju W‐K (2018) Elevated intracellular cAMP exacerbates vulnerability to oxidative stress in optic nerve head astrocytes article. Cell Death Dis 9, 285. 29459737 10.1038/s41419-017-0171-8 PMC5833440

[91] Luu J , Saadane A , Leinonen H , Luu JC , Choi EH , Gao F , Lewandowski D , Halabi M , Sander CL , Wu A *et al*. (2023) Stress resilience‐enhancing drugs preserve tissue structure and function in degenerating retina via phosphodiesterase inhibition. Proc Natl Acad Sci U S A 120, e2221045120. 37126699 10.1073/pnas.2221045120 PMC10175720

[92] Cohen A , Toddt R , Harmont S and O'malleyt KL (1992) Photoreceptors of mouse retinas possess D4 receptors coupled to adenylate cyclase. Proc Natl Acad Sci U S A 89, 12093–12097. 1334557 10.1073/pnas.89.24.12093 PMC50704

[93] Goel M and Mangel SC (2021) Dopamine‐mediated circadian and light/dark‐adaptive modulation of chemical and electrical synapses in the outer retina. Front Cell Neurosci 15, 647541. 34025356 10.3389/fncel.2021.647541 PMC8131545

[94] Popova E (2020) Role of dopamine in retinal function. In Webvision: The Organization of the Retina and Visual System ( Kolb H , Fernandez E , Jones B and Nelson R , eds), University of Utah Health Sciences Center, Salt Lake City, UT.

[95] Klitten L , Rath M , Coon S , Klitten LL , Rath MF , Coon SL , Kim JS , Klein DC and Møller M (2008) Localization and regulation of dopamine receptor D4 expression in the adult and developing rat retina. Exp Eye Res 87, 471–477. 18778704 10.1016/j.exer.2008.08.004 PMC2597030

[96] Deming J , Shin JA , Lim K , Deming JD , Lee E‐J , Van Craenenbroeck K and Craft CM (2015) Dopamine receptor D4 internalization requires a beta‐arrestin and a visual arrestin. Cell Signal 27, 2002–2013. 26169958 10.1016/j.cellsig.2015.06.008 PMC9112046

[97] Jackson C , Chaurasia S , Zhou H , Jackson CR , Chaurasia SS , Haque R , Storm DR and Iuvone PM (2009) Essential roles of dopamine D4 receptors and the type 1 adenylyl cyclase in photic control of cyclic AMP in photoreceptor cells. J Neurochem 109, 148–157. 19166506 10.1111/j.1471-4159.2009.05920.x PMC2727872

[98] Liu W , Ha Y , Xia F , Zhu S , Li Y , Shi S , Mei FC , Merkley K , Vizzeri G , Motamedi M *et al*. (2020) Neuronal Epac1 mediates retinal neurodegeneration in mouse models of ocular hypertension. J Exp Med 217, e20190930. 31918438 10.1084/jem.20190930 PMC7144517

[99] Allison A (2005) Mechanisms of action of mycophenolate mofetil. Lupus 14, S2–S8. 15803924 10.1191/0961203305lu2109oa

[100] Daniel E , Thorne J , Newcomb C , Pujari SS , Kaçmaz RO , Levy‐Clarke GA , Nussenblatt RB , Rosenbaum JT , Suhler EB , Foster CS *et al*. (2010) Mycophenolate mofetil for ocular inflammation. Am J Ophthalmol 149, 422–423. 10.1016/j.ajo.2009.09.026 PMC2826576 20042178

[101] Farzam K and Jan A (2023) Beta Blockers. StatPearls, Treasure Island, FL. 30422501

[102] Harada K , Ohmori M and Fujimura A (1996) Comparison of the antagonistic activity of tamsulosin and doxazosin at vascular alpha 1‐adrenoceptors in humans. Naunyn Schmiedebergs Arch Pharmacol 354, 557–561. 8938652 10.1007/BF00170828

[103] del Amo E , Rimpelä AK , Heikkinen E , Kari OK , Ramsay E , Lajunen T , Schmitt M , Pelkonen L , Bhattacharya M , Richardson D *et al*. (2017) Pharmacokinetic aspects of retinal drug delivery. Prog Retin Eye Res 57, 134–185. 28028001 10.1016/j.preteyeres.2016.12.001

[104] Ramsay E , Lajunen T , Bhattacharya M , Reinisalo M , Rilla K , Kidron H , Terasaki T and Urtti A (2023) Selective drug delivery to the retinal cells: biological barriers and avenues. J Control Release 361, 1–19. 37481214 10.1016/j.jconrel.2023.07.028

[105] Samoilă L , Voștinaru O , Dinte E , Bodoki AE , Iacob BC , Bodoki E and Samoilă O (2023) Topical treatment for retinal degenerative pathologies: a systematic review. Int J Mol Sci 24, 8045. 37175752 10.3390/ijms24098045 PMC10178888

[106] Lanier O , Manfre M , Mailey C , Liu Z , Sparks Z , Kulkarni S and Chauhan A (2021) Review of approaches for increasing ophthalmic bioavailability for eye drop formulations. AAPS PharmSciTech 22, 107. 33719019 10.1208/s12249-021-01977-0

[107] Joussen A , Wolf S , Kaiser P , Joussen AM , Kaiser PK , Boyer D , Schmelter T , Sandbrink R , Zeitz O , Deeg G *et al*. (2018) The developing regorafenib eye drops for neovascular age‐related macular degeneration (DREAM) study: an open‐label phase II trial. Br J Clin Pharmacol 85, 347–355. 30341774 10.1111/bcp.13794 PMC6339971

[108] Samanta A, Aziz AA, Jhingan M, Singh SR, Khanani AM, Chhablani J (2020) Emerging Therapies in Neovascular Age‐Related Macular Degeneration in 2020. Asia Pac J Ophthalmol 9 , 250–259. 10.1097/APO.0000000000000291 PMC7299216 32511123

[109] Study of PAN‐90806 Eye Drops, Suspension for Neovascular AMD. Clinicaltrials.gov, NCT03479372.

[110] Bakthavatchalam M , Lai F , Rong S , Ng D and Brelen M (2018) Treatment of cystoid macular edema secondary to retinitis pigmentosa: a systematic review. Surv Ophthalmol 63, 329–339. 28987613 10.1016/j.survophthal.2017.09.009

[111] Gaudana R , Ananthula H , Parenky A and Mitra A (2010) Ocular drug delivery. AAPS J 12, 348–360. 20437123 10.1208/s12248-010-9183-3 PMC2895432

[112] Kim H and Woo S (2021) Ocular drug delivery to the retina: current innovations and future perspectives. Pharmaceutics 13, 108. 33467779 10.3390/pharmaceutics13010108 PMC7830424

[113] Molokhia S , Papangkorn K , Butler C , Higuchi JW , Brar B , Ambati B , Li SK and Higuchi WI (2020) Transscleral iontophoresis for noninvasive ocular drug delivery of macromolecules. J Ocul Pharmacol Ther 36, 247–256. 32155098 10.1089/jop.2019.0081 PMC7232671

[114] Park J , Zhang Y , Vykhodtseva N , Akula J and McDannold N (2012) Targeted and reversible blood‐retinal barrier disruption via focused ultrasound and microbubbles. PLoS One 7, e42754. 22912733 10.1371/journal.pone.0042754 PMC3418291

[115] Tawfik M , Chen F , Goldberg J and Sabel B (2022) Nanomedicine and drug delivery to the retina: current status and implications for gene therapy. Naunyn Schmiedebergs Arch Pharmacol 395, 1477–1507. 36107200 10.1007/s00210-022-02287-3 PMC9630211

